# The complete chloroplast genome of *Ishige okamurae*

**DOI:** 10.1080/23802359.2021.1950583

**Published:** 2021-07-19

**Authors:** Xixi Wang, Changfeng Qu, Jianjun Dong, Jinlai Miao

**Affiliations:** a State Key Laboratory of Biological Fermentation Engineering of Beer, Qingdao, China; b Ministry of Natural Resources, First Institute of Oceanography, Qingdao, China; c College of Chemistry and Molecular Engineering, Qingdao University of Science and Technology, Qingdao, China; d Laboratory for Marine Drugs and Bioproducts, Qingdao National Laboratory for Marine Science and Technology, Qingdao, China

**Keywords:** *Ishige okamurae*, chloroplast genome, phylogenetic analysis

## Abstract

*Ishige okamurae* plays an important role in marine ecosystem and biological resource utilization. The total length of chloroplast genome was 129,988 bp, containing a large single-copy region (LSC, 77,531 bp), a small single-copy region (SSC, 41,795 bp) and a pair of inverted repeat regions (IRs, 10,662 bp). The circular genome consisted of 101 protein-coding genes, 29 tRNA genes, and six rRNA genes, with a total of 136. Phylogenetic analysis confirmed the position of *I. okamurae* within the Phaeophyta.

*Ishige okamurae* belongs to Phaeophyceae, Chordariales, Ishigeaceae, Ishige, and is a representative species of this genus (Lee et al. 2009; Sanjeewa et al. 2017). It can adapt to complex intertidal habitats and maintain basal metabolism in extremely harsh environments. The biological characteristics and distribution of *I. okamurae* are unique, making it be an excellent object of phylogeography (Lee et al. 2013). In recent years, *I. okamurae* has aroused widespread attention and exploration of scientific researchers in the development of marine ecosystems and active substances.

The research objects used in this experiment were collected from the Banzi Jiao (24°42′ N, 118°12′ E) of Fujian Province and preserved in the first Institute of Oceanography, Ministry of natural resources (Accession number: FIO2019091201). Shanghai Biozeron Biological Technology Co. Ltd implemented the construction of the whole genome library of *I. okamurae* chloroplast. The genomic paired-end sequencing was carried out on the Illumina NovaSeq 6000 platform. Since the original sequencing data of Illumina had some low-quality data, in order to make the subsequent assembly more accurate, the original data (4851.6 Mb) were filtered and evaluated. Subsequently, the chloroplast genome was assembled using NOVOPlasty v4.2 software and GeSeq software to predict the functional genes (Dierckxsens et al. 2017; Tillich et al. 2017). The genomic sequence had been submitted to GenBank with accession number MW762687.

The results showed that the chloroplast genome size was 129,988 bp, and the predicted number of coding genes was 136. The total length of coding genes was 94,212 bp, accounting for 72.48% of the total length. The circular genome consisted of a large single-copy region (LSC, 77,531 bp), a small single-copy region (SSC, 41,795 bp) and a pair of inverted repeat regions (IRs, 10,662 bp). The chloroplast genome composition was 32.51% A, 34.01% T, 16.24% G, 17.24% C, with the G + C content accounts for 33.48%. Among the coding genes, there were 101 protein-coding genes, 29 tRNA genes, and six rRNA genes.

To further investigate the phylogenetic relationship of *I. okamurae*, we used MEGA 7.0 to perform multiple sequence alignment with the chloroplast genomes of 10 species selected from NCBI-GenBank database and constructed a maximum-likelihood (ML) phylogenetic tree (Figure 1) (Kumar et al. 2016). The nucleotide substitution model is General Time Reversible (GTR, nst = 6) here (Rodríguez et al. 1990). The phylogenic result demonstrated that *I. okamurae* was closely related with *Dictyopteris divaricate*.

**Figure 1.**
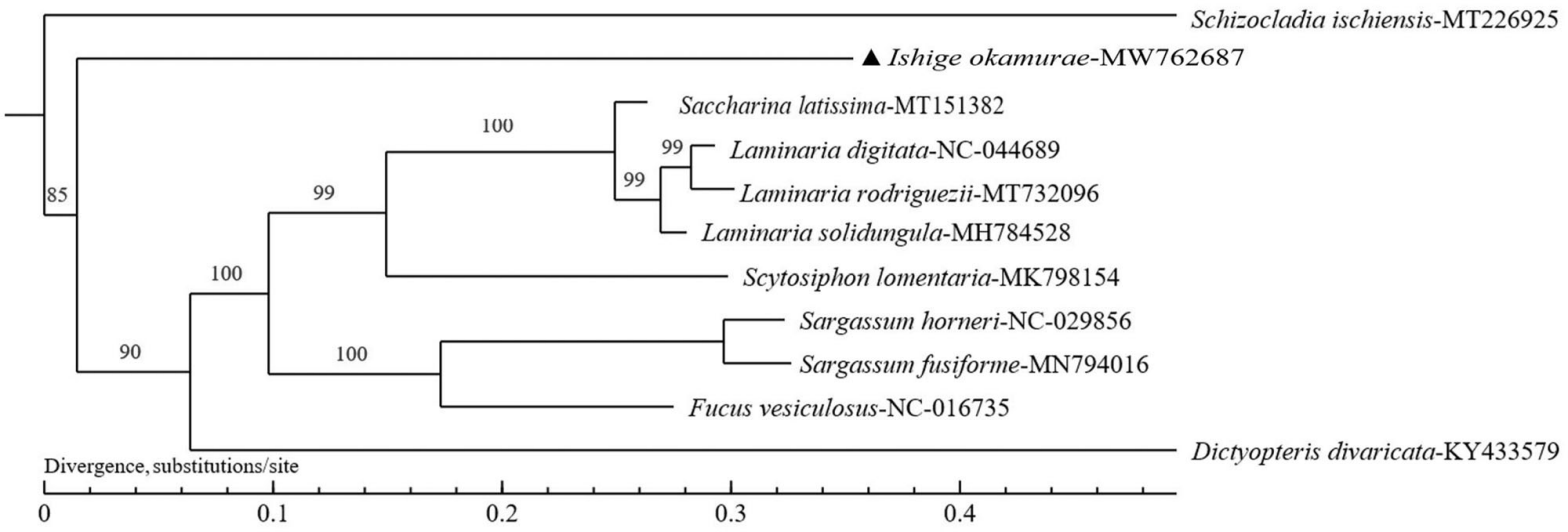
Phylogenetic tree of *I. okamurae* and 10 species constructed using the ML method based on chloroplast genome sequences. Numbers on the nodes were bootstrap values from 1000 replicates. Schizocladia ischiensis was selected as an outgroup.

## Data Availability

The data that support the findings of this study are openly available in the NCBI at https://www.ncbi.nlm.nih.gov/, reference number of MW762687.
